# Catalase Modulates the Radio-Sensitization of Pancreatic Cancer Cells by Pharmacological Ascorbate

**DOI:** 10.3390/antiox10040614

**Published:** 2021-04-16

**Authors:** Juan Du, Rory S. Carroll, Garett J. Steers, Brett A. Wagner, Brianne R. O’Leary, Chris S. Jensen, Garry R. Buettner, Joseph J. Cullen

**Affiliations:** 1 Free Radical and Radiation Biology Program, Department of Radiation Oncology, The University of Iowa Carver College of Medicine, Iowa City, IA 52242, USA; juan-du@uiowa.edu (J.D.); rory-carroll@uiowa.edu (R.S.C.); garett-steers@uiowa.edu (G.J.S.); brianne-oleary@uiowa.edu (B.R.O.); 2 Department of Surgery, The University of Iowa Carver College of Medicine, Iowa City, IA 52242, USA; brett-wagner@uiowa.edu (B.A.W.); garry-buettner@uiowa.edu (G.R.B.); 3 Department of Pathology, The University of Iowa Carver College of Medicine, Iowa City, IA 52242, USA; chris-jensen@uiowa.edu

**Keywords:** pharmacological ascorbate, DNA damage, DNA repair, pancreatic cancer, vitamin C

## Abstract

Pancreatic cancer cells (PDACs) are more susceptible to an oxidative insult than normal cells, resulting in greater cytotoxicity upon exposure to agents that increase pro-oxidant levels. Pharmacological ascorbate (P-AscH^−^), i.e., large amounts given intravenously (IV), generates significant fluxes of hydrogen peroxide (H_2_O_2_), resulting in the killing of PDACs but not normal cells. Recent studies have demonstrated that P-AscH^−^ radio-sensitizes PDAC but is a radioprotector to normal cells and tissues. Several mechanisms have been hypothesized to explain the dual roles of P-AscH^−^ in radiation-induced toxicity including the activation of nuclear factor-erythroid 2-related factor 2 (Nrf2), RelB, as well as changes in bioenergetic profiles. We have found that P-AscH^−^ in conjunction with radiation increases Nrf2 in both cancer cells and normal cells. Although P-AscH^−^ with radiation decreases RelB in cancer cells vs. normal cells, the knockout of RelB does not radio-sensitize PDACs. Cellular bioenergetic profiles demonstrate that P-AscH^−^ with radiation increases the ATP demand/production rate (glycolytic and oxidative phosphorylation) in both PDACs and normal cells. Knocking out catalase results in P-AscH^−^ radio-sensitization in PDACs. In a phase I trial where P-AscH^−^ was included as an adjuvant to the standard of care, short-term survivors had higher catalase levels in tumor tissue, compared to long-term survivors. These data suggest that P-AscH^−^ radio-sensitizes PDACs through increased peroxide flux. Catalase levels could be a possible indicator for how tumors will respond to P-AscH^−^.

## 1. Introduction

Adenocarcinoma of the pancreas (PDAC) is the fourth leading cause of cancer death in the U.S. [1]. While surgery can be used as a local therapy, the majority of patients have disease that is too advanced to allow for resection. The alternative local therapy is to utilize ionizing radiation either alone or in combination with chemotherapy. Despite efforts to improve treatment efficacy by increasing the intensity of treatment by altering radiation fractionation or with different concurrent chemotherapies, one-third of patients will experience local failure and succumb to their disease [2]. Thus, there are a significant number of PDAC patients who would benefit from improvements in the efficacy of standard of care. However, the use of curative doses of radiation in this disease setting is constrained due to the close proximity of the head of the pancreas to the duodenum, a radiosensitive organ. A potential approach to bypass this limitation is to develop methods to provide protection of the duodenum from radiation treatment regimens. Protecting normal tissue (duodenum) against damage from high-dose radiation would allow dose escalation, leading to the enhanced killing of PDAC.

There is a fundamental difference in the redox environment of normal cells compared to tumor cells; tumor cells have a higher steady-state level of superoxide and related oxidants compared to normal cells [3,4,5,6]. Thus, as such, treatments that are designed to increase oxidative distress are anticipated to enhance toxicity for cancer cells, whereas normal cells with a more efficient antioxidant network and a lower level of oxidants are anticipated to be more resistant to such oxidative insults. Pharmacological ascorbate (P-AscH^−^, 5–100 g given intravenously) is a prototypical antioxidant/pro-oxidant that can elicit a protective response in normal cells but add injury to tumor cells. Our in vitro, in vivo, [7,8,9] and human (phase I trials; NCT 01049880, 01852890; PI: Cullen) studies [6,7,8,9,10] have shown that P-AscH^−^-mediated cell death is due to the generation of H_2_O_2_, with ascorbate as the electron donor to dioxygen [3]. P-AscH^−^ has been shown to exert the majority of its pro-oxidant effects by the extracellular generation of H_2_O_2_ [8,11,12,13,14,15]. Compared to the oral administration of ascorbate, plasma concentrations achieved by intravenous delivery can be very high, 10–30 mM, compared to a maximum of about 0.2 mM with oral delivery; IV delivery is safe, well tolerated, and shows potential efficacy as an adjuvant to some cancer therapies [6,9].

Our studies have demonstrated that P-AscH^−^ is the prototypical antioxidant/pro-oxidant that can elicit a protective response in normal cells but inflict injury to tumor cells after radiation therapy [6,16]. P-AscH^−^ enhanced the cytotoxic effects of radiation, as seen by decreased cell viability and clonogenic survival in all PDAC cell lines examined, but not in non-tumorigenic pancreatic ductal epithelial cells. P-AscH^−^ radio-sensitization was associated with an increase in oxidative stress-induced DNA damage, which was reversed by catalase. In mice with established heterotopic and orthotopic PDAC xenografts, P-AscH^−^ combined with radiation decreased tumor growth and increased survival. Most importantly, P-AscH^−^ reversed radiation-induced damage to the gastrointestinal tract but did not increase systemic changes in parameters indicative of oxidative stress. The potential clinical utility of P-AscH^−^ as an adjuvant to standard of care therapy for PDAC led us to the first in human phase I trial, demonstrating that P-AscH^−^ in combination with gemcitabine and radiation for locally advanced PDAC is safe and well tolerated with increases in progression-free and overall survival [7].

The mechanism by which P-AscH^−^ elicits a protective response in normal cells but increases toxicity to tumor cells is under intense investigation. To elucidate the mechanism on how P-AscH^−^ radio-sensitizes tumors but radio-protects normal cells, we examined the effects of P-AscH^−^ and radiation on nuclear factor-erythroid 2-related factor 2 (Nrf2), RelB, and the bioenergetic profiles in several PDAC cell lines as well as normal cells. In addition, we provide follow-up on three long-term survivors from a phase I trial in which P-AscH^−^ was administered during chemo-radiation therapy.

## 2. Materials and Methods

### 2.1. Cell Culture

All cell lines were purchased from American Type Culture Collection (ATCC). MIA PaCa-2 and PANC-1 human PDAC cells were cultured in Dulbecco’s Modified Eagle’s Media (DMEM) with high glucose, supplemented with 10% fetal bovine serum (FBS) and penicillin (100 units mL^−1^)/streptomycin (100 µg mL^−1^). The human non-tumorigenic, immortalized pancreatic ductal epithelial cells, H6c7, were cultured in keratinocyte serum-free media, supplemented with epidermal growth factor (5 ng mL^−1^), bovine pituitary extract (50 μg mL^−1^), and penicillin (100 units mL^−1^) with streptomycin (100 µg mL^−1^). Human fetal small intestine cells FHs74 Int (human fetal small intestine cells) were cultured in Hybri-Care Medium ATCC 46-X supplemented with 30 ng mL^−1^ epidermal growth factor (EGF) and 10% FBS. All cells were cultured at 37 °C in a humidified atmosphere of 95% air/5% CO_2_.

### 2.2. Treatment of Cells with P-AscH^−^

L-ascorbic acid was obtained from Macron Fine Chemicals. Stock solutions of L-ascorbic acid (1.0 M, pH 7.0) were prepared under argon and stored in screw-cap glass vials at 4 °C. The formation of H_2_O_2_ due to the oxidation of P-AscH^−^ is dependent on the pH and iron components of media [17]. Hence, cells were always treated in DMEM–10% FBS for 1 h to minimize differences in pH and iron components and thereby minimizing the differences in the flux of H_2_O_2_ between experiments [7]. The dose of ascorbate is best specified as moles per cell (here pmol cell^−1^) [18,19]. After treatment, the cells were trypsinized for clonogenic survival assay or cultured in their growth media for different time points before harvesting for Western blot analysis.

### 2.3. Western Blot Analysis

Live cells were isolated and lysed in RIPA buffer and pelleted by centrifugation. Protein concentrations were determined using a Bio-Rad DC Bradford Protein Assay (Bio-Rad Laboratories, Hercules, CA, USA). 40 μg of protein were electrophoresed in a Bio-Rad 4–20% Precast Gel for 65 min at 120 V. Proteins were electro-transferred onto a polyvinylidene difluoride (PVDF) membrane (Millipore, Burlington, MA, USA), and blocked with 5% nonfat milk in 0.1% Tween-PBS (TPBS) for 60 min. The membranes were incubated with primary antibodies (1:1000, Cell Signaling Technology, Danvers, MA, USA) at 4 °C overnight. Membranes were washed 5 times with TPBS and incubated with secondary antibodies conjugated with horseradish peroxidase (1:20,000, Millipore, Temecula, CA, USA). GAPDH (1:5000, Millipore) or tubulin (1:2000, Cell Signaling) was used as a loading control. After being washed with TPBS, membranes were stained with Super Signal West Pico Chemiluminescent Substrate (Thermo Scientific, Rockford, IL, USA) and exposed to Classic Blue Autoradiography Film (Molecular Technologies, St Louis, MO, USA).

### 2.4. Generation of RelB or Catalase CRISPR/Cas9 Knockout Cells

MIA PaCa-2 or PANC-1 cells were seeded into 6-well plates at 150,000 cells in 3.0 mL of media and were cultured at 37 °C, 5% CO_2_ for 24 h. Standard medium was replaced with 3.0 mL of antibiotic-free DMEM prior to transfection. After media replacement, cells were then transfected with RelB CRISPR/Cas9 KO plasmid (Santa Cruz Biotechnology, Inc.; Dallas, TX, USA; cat. No. sc-422643) or Catalase CRISPR/Cas9 KO plasmid (Santa Cruz Biotechnology, cat. No. sc-400353) for 24 h. After transfection, cells were grown in growth media for an additional 48 h. To create a single monoclonal cell line with RelB or catalase knockout, the GFP-positive cells were selected by using BD FACs Aria Fusion flow cytometer (Becton Dickinson; Franklin Lakes, NJ, USA) at the Flow Cytometry Facility of the University of Iowa; single cells were sorted into individual wells of a 96-well plate. The cell lines with bi-allelic or mono-allelic RelB or catalase knockouts were determined with Western blot analysis. Using similar procedures, control cells were generated by transfecting parental cell lines with control CRISPR/Cas 9 plasmid (Santa Cruz Biotechnology, Inc.; Dallas, TX, USA; cat. No. sc-418922).

### 2.5. Catalase Activity Assay

Three million cells grown on 100-mm tissues culture dishes were detached by 0.25% trypsin-ethylenediaminetetraacetic acid (trypsin-EDTA) and the cell pellets were stored at −80 °C. For an assay, 70 microliters of phosphate buffer (50 mM, pH 7.0) were added to the cell pellet and sonicated for 30 s using a Vibra-Cell sonicator (Sonics, Newton, CT). After sonication, to break up cell membranes, samples were centrifuged for 5 min at 10,000 *g* in an Eppendorf Microcentrifuge. Five hundred microliters of phosphate buffer (50mM, pH 7.0) were added to a 10 mm pathlength quartz cuvette (Starna Cells, Atascadero, CA, USA), followed by 20 µL of cell lysate and 250 µL of 30 mM H_2_O_2_. Catalase activity were measured in a HP UV–Vis Spectrophotometer. The absorbance at 240 nm was followed for 120 s with a cycle time of 10 s. The activity of catalase in cells was determined as m*k*Units per million cells [20,21].

### 2.6. Clonogenic Assay

Cells were seeded into multiple 60 mm^2^ culture dishes at 100,000 cells per dish in 4.0 mL of medium. Cells were cultured in their respective media for 48 h at 37 °C, 5% CO_2_. Following treatment, exposure media were removed; cells were trypsinized, counted using a Moxi Z Mini Automated Cell Counter (ORFLO Technologies; Ketchum, ID, USA), and seeded in triplicate into 6-well plates at 300 cells per well in 4.0 mL of their respective medium.

### 2.7. Measurement of Cellular Metabolic Flux

MIA PaCa-2 cells were seeded into the Seahorse 96-well XF Cell Culture Microplate V3-PS (Agilent Technologies, Inc.; Santa Clara, CA, USA) at 20,000 cells per well in 200 μL of growth media, FHs74Int cells at 40,000 cells per well. After 48 h, half of the plate were irradiated at 5 Gy; the other half at 5 Gy, then treated with P-AscH^−^ (5 pmol cell^−1^, 2 mM for 1 h). After treatment, fresh growth media were added, then plates were incubated for 48 h. The cells were washed with Seahorse media (pre-warmed XF Base Medium Minimal DMEM containing 10 mM glucose, 2 mM glutamine, 5mM HEPES, and 1 mM sodium pyruvate), with the pH of media approximately 7.4 and buffer capacity determined prior to each assay. Oxygen consumption rate (OCR, i.e., flow per cell *I_O_*_2*/cell*_) was then measured using the Seahorse XF96 analyzer with the standard mitochondrial stress test. At the end of experiments, the cell density from representative wells was determined with Moxi cell counter. Using the average cell density across the wells of a certain condition, the OCR was normalized per cell, which is considered as flow *I_O_*_2*/cell*_. The basal OCR was determined from the parameters obtained from the mitochondrial stress test; OCR_basal_ = OCR_ATP_ + OCR_proton leak_.

### 2.8. Confocal Microscopy (Catalase Immunofluorescence)

Formalin-fixed, paraffin-embedded tissue samples were cut with Thermo HM525 Cryostat (Thermo Scientific). Tissue sections were deparaffinized, processed in antigen unmask solution and washed with PBS before blocking with 5% normal goat serum for 30 min. Sections were incubated with rabbit catalase antibody (1:50, Cell Signaling, 14079S) in 1% NGS overnight at 4 °C. An Alexa Flour 488 conjugated goat anti-rabbit (1:200) was used as the secondary antibody. DAPI was used to stain the cell nuclei. Tumor tissue sections were examined with a Zeiss LSM710 confocal microscope (Central Microscopy Research Facility, University of Iowa). The intensity of catalase immunofluorescence was quantified using ImageJ.

### 2.9. Follow-Up of Phase I Trial Long Term Survivors

The clinical trial “Gemcitabine, Ascorbate, Radiation therapy for PDAC, Phase I” (NCT01852890) was a single-institution, open-label phase I study performed at the University of Iowa. Approved by the FDA and the University of Iowa IRB-01, the P-AscH^−^ dose was escalated via Storer’s Phase I Two-Stage Design BD [22]. There were three planned cohorts: 50 g, 75 g, and 100 g. Patients were required to have histologically or cytologically diagnosed PDAC and indication for chemoradiotherapy. Gemcitabine was administered with an infusion at a dose of 600 mg/m^2^ over 30 min, once weekly for 6 weeks [23]. Patients were treated with either 50.4 Gy in 28 fractions or 50 Gy in 25 fractions. Fourteen patients completed all protocol therapy. No subjects were lost to follow-up. A comparator cohort of 21 patients at our institution who received gemcitabine and ionizing radiation (IR) were also evaluated. The electronic medical records of three long term survivors were reviewed for evidence of recurrence. Overall survival and progression-free survival curves from the original data of 14 treatment subjects and 19 comparators were updated to reflect ongoing survival using GraphPad Prism 8 software.

## 3. Results

### 3.1. Activation of Nuclear Factor-Erythroid 2-Related Factor 2 Nrf2

**Figure 1:**
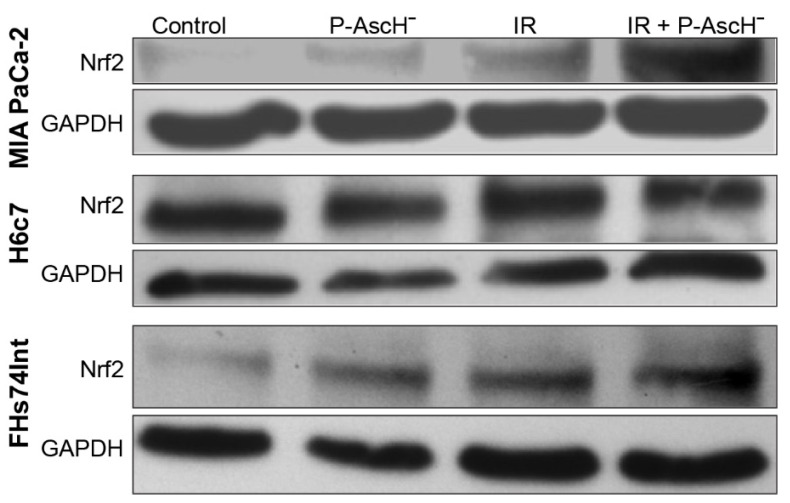
Changes in nuclear factor-erythroid 2-related factor 2 (Nrf2) activation of normal cells vs. pancreatic cancer cells (PDACs) after pharmacological ascorbate (P-AscH^−^), radiation, or the combination of both. Western blot analyses show that P-AscH^−^, radiation, or radiation + P-AscH^−^ induce Nrf2 in MIA PaCa-2 and FHs74Int cells, but no change occurs in H6c7 cells. Cells were treated with P-AscH^−^ (5 pmol/cell, 1 mM) for 1 h, 5 Gy, or 5 Gy + P-AscH^−^ in DMEM−10% fetal bovine serum (FBS). After treatment, cells were cultured in their growth media for 24 h before protein was harvested for Western blot analysis.

Cancer cells are more sensitive than normal cells to the toxic species generated by P-AscH^−^ and radiation [24]. Transcription mediated by nuclear factor-erythroid 2-related factor 2 (Nrf2) can protect cells and tissues from the pathological consequences of reactive oxygen species including hydroxyl radicals that are directly generated by ionizing radiation as well as the hydrogen peroxide and superoxide that are generated as a secondary consequence of irradiation [25]. Recently, the activation of redox sensitive pro-survival transcription factor Nrf2 was shown to protect colonic epithelial cells from radiation-induced loss in cell viability [26]. P-AscH^−^, radiation, and P-AscH^−^ + radiation activated Nrf2 to a similar degree in the PDAC cell line MIA PaCa-2 and FHS74Int cells, increasing levels to those similar in the normal cell line, H6c7 (Figure 1). These data suggest that Nrf2 activation may promote the survival of both PDAC cells and normal cells equivalently but may not have a major role in P-AscH^−^ radio-sensitization of PDAC and the radio-protection of normal cells.

### 3.2. RelB in Radiation and P-AscH^−^ Response in PDAC

**Figure 2:**
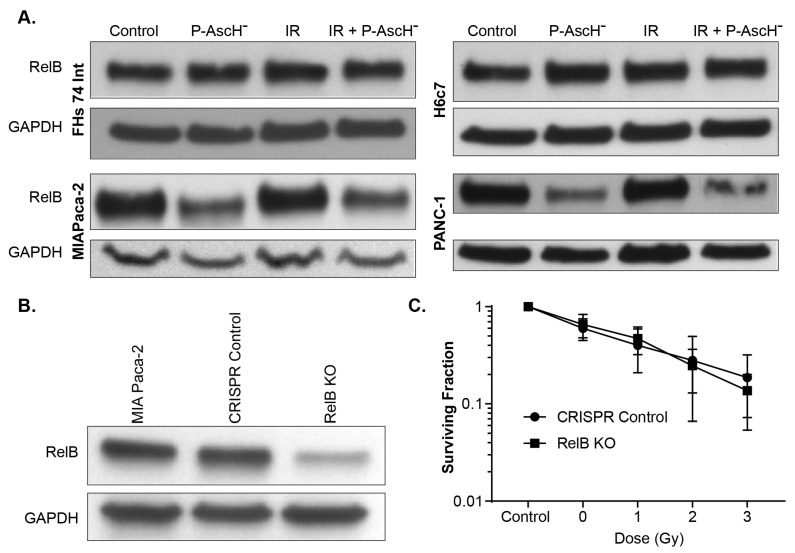
Role of RelB in the differential response of normal and PDAC cells. (**A**) Cells were treated with P-AscH^−^: MIA PaCa-2, FHs74Int, and H6c7 with 5 pmol cell^−1^ (1 mM) and PANC-1 with 15 pmol cell^−1^ (3 mM); radiation (5 Gy); or the combination of both. P-AscH^−^ with or without radiation, decreases RelB levels in MIA PaCa-2 and PANC-1 cells, but not in FHs74Int and H6c7 cells; (**B**) CRISPR/Cas9 KO plasmid was used to generate RelB knockout cells in MIA PaCa-2 PDAC (RelB KO). Control cells (CRISPR control) were generated by transfecting parental cell lines with control CRISPR/Cas 9 plasmid, resulting in decreased immunoreactive protein in the RelB KO; (**C**) clonogenic survival. Treatment of PDAC CRISPR control and RelB KO with P-AscH⁻ (5 pmol cell^−1^, 1 mM) and IR (1–3 Gy), showed no differences in clonogenic survival between the two groups.

Reactive oxygen species (ROS) are known inducers of nuclear factor-κB (NFκB). The NFκB subunit RelB plays an important role in the radio-resistance of prostate cancer. The expression of RelB in normal and prostate cancer cells serves as a central regulator for their opposing responses to radiotherapy [27]. Wei et al. found that the suppression of RelB decreases the expression of the sirtuin SIRT3 and MnSOD (SOD2), which in turn increases oxidative and metabolic stress in prostate cancer cells [26]. In contrast, our results demonstrate that P-AscH^−^ or P-AscH^−^ and radiation decrease RelB levels in the PDAC MIA PaCa-2 and Panc-1 cells, but not in human fetal small intestine cells FHs74Int and immortalized pancreatic ductal epithelial cells H6c7 (Figure 2A). Wei and colleagues also demonstrated that the inhibition of RelB in prostate cancer cells decreased cell viability. To further define the role of RelB in the different responses of cancer cells vs. normal cells against radiation or radiation with P-AscH^−^ treatment, we used RelB CRISPR/Cas9 KO plasmid in MIA PaCa-2 cells to generate RelB knockout cells (MIA RelB KO). Using similar procedures, control cells were generated by transfecting parental cell lines with control CRISPR/Cas 9 plasmid (Figure 2B). When MIA RelB KO and CRISPR control cells exposed to P-AscH^−^ (5 pmol cell^−1^, 1 mM) or radiation 1, 2 and 3 Gy with P-AscH^−^, there was no significant difference in clonogenic survival between the two groups (two-way ANOVA, *p* > 0.05) (Figure 2C). Thus, our data suggest that RelB does not play a significant role in radio-sensitization by P-AscH^−^.

### 3.3. No Changes in Bioenergetics in Normal and PDAC Cells after Exposure to P-AscH^−^ and Radiation

**Figure 3:**
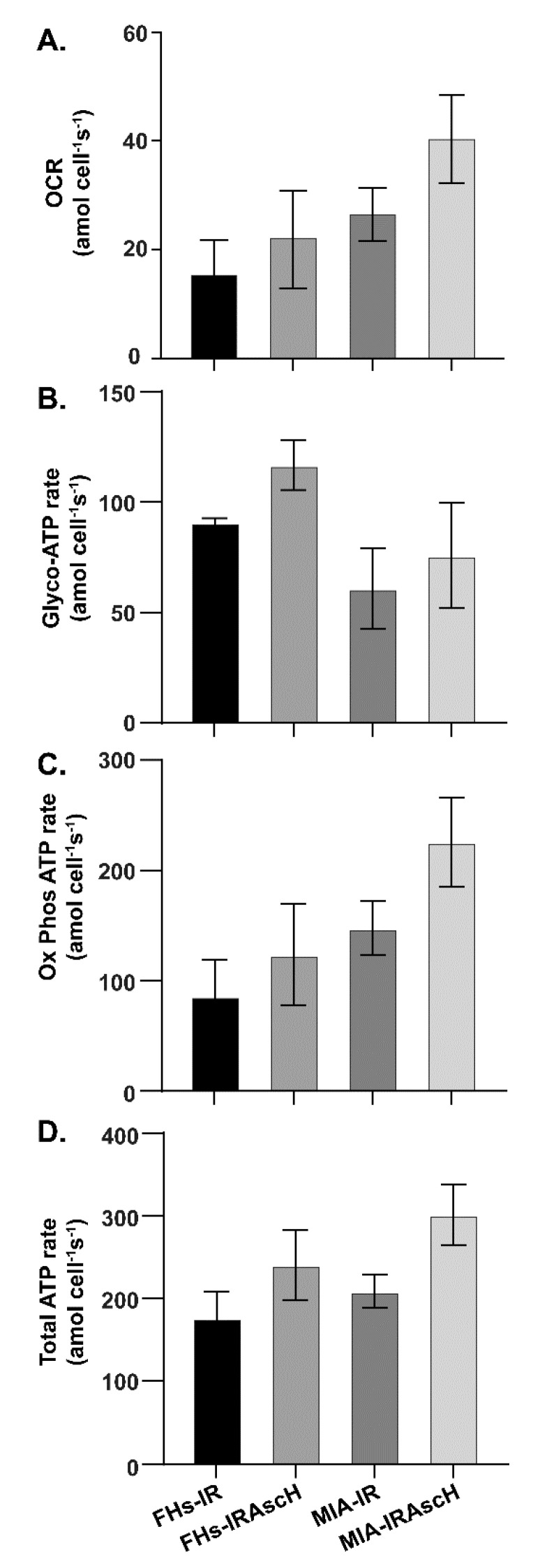
Bioenergetics after treatment with P-AscH^⁻^ and radiation. In both normal and PDAC cell lines, P-AscH^−^ (5 pmol cell^−1^, 1 mM) with and without radiation (5 Gy) results in increased rate of basal oxygen consumption (OCR) and increased rate of production of ATP 48 h after treatment. (**A**) MIA PaCa-2 and FHs74Int cells were treated with P-AscH^−^ for 1 h and then basal oxygen consumption rate (OCR, i.e., flow per cell *I_O_*2*_/cell_*) was measured 48 h after treatment; (**B**) total ATP changes were similar in FHs74Int vs. MIA PaCa-2 48 h after radiation with or without P-AscH^−^. The contributions to the changes in ATP were then determined using Seahorse XF96 instrumentation; (**C**) ATP rate attributed to oxidative phosphorylation demonstrated similar changes in FHs74Int cells vs. MIA PaCa-2 cells 48 h after radiation with or without P-AscH^−^; (**D**) ATP rate attributed to glycolysis demonstrated similar changes in in FHs74Int cells vs. MIA PaCa-2 cells 48 h after radiation with or without P-AscH^−^. (Means ± SEM, *n* = 3. There were no significant differences between any of the treatment groups).

Previous studies have shown that P-AscH^−^ depletes cellular ATP via the formation of H_2_O_2_ in PDAC cells following 1-h exposure [28]. However, the rapid reduction in energy stores upon exposure to P-AscH^−^ was due to increased demand in response to DNA damage repair and was not caused by changes in the rate of production from respiration or glycolysis. On the contrary, 48 h after P-AscH^−^ treatment, there were increases in basal OCR, ATP-linked OCR, maximal respiration and proton leak in pancreatic cancer cells [9]. Thus, we investigated possible changes in the bioenergetic profile following radiation (5 Gy) with and without P-AscH^−^ in both the FHs74Int cell line and MIA PaCa-2 cells. Radiation increased ATP-linked OCR, i.e., the rate of production from oxidative phosphorylation. The overall rate of ATP production was further increased upon the addition of P-AscH^−^ (5 pmol cell^−1^, 1 mM) (Figure 3). This occurred to a similar degree in both the intestinal cells and the PDAC cell line, suggesting that radiation-induced changes in OCR were increased with the addition of P-AscH^−^, but tumor and normal cells behaved similarly.

### 3.4. Catalase Affects Radiosensitization

**Figure 4:**
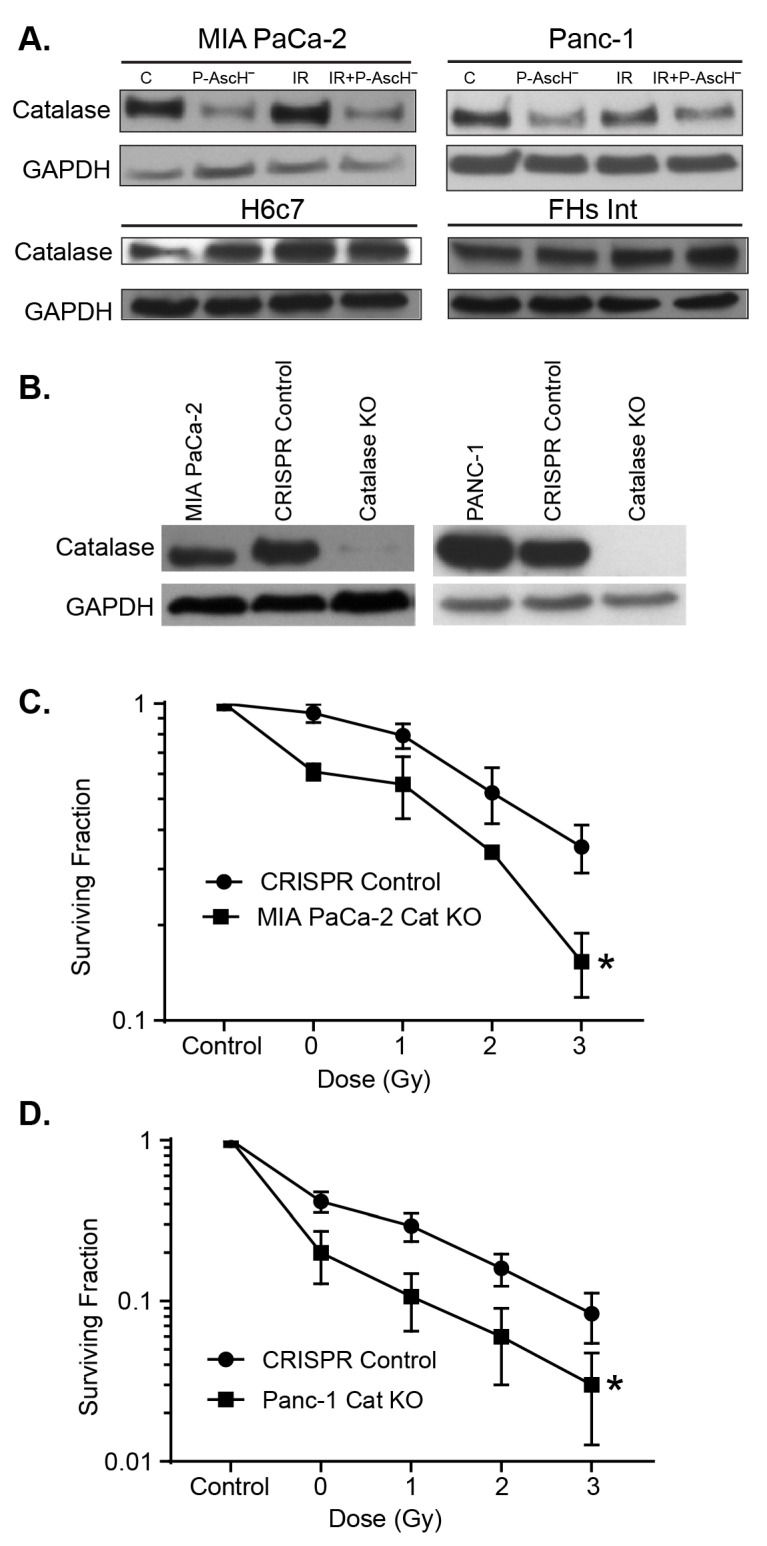
Cellular catalase blunts the radio-sensitization of P-AscH^−^. (**A**) The PDAC cell lines MIA PaCa-2 (5 pmol cell^−1^, 1 mM) and PANC-1 (15 pmol cell^−1^, 3 mM), and the non-tumorigenic H6c7 and FHs74Int cell lines were treated with P-AscH^−^ (5 pmol cell^−1^, 1 mM) with and without IR (5 Gy). Live cells were collected and Western blots performed to determine catalase immunoreactive protein. P-AscH^−^ alone and in combination with radiation, decreased catalase in the PDAC cell lines, but not in the H6c7 or FHsInt cell lines; (**B**) clones of MIA PaCa-2 and PANC-1 PDAC cells subjected to CRISPR/Cas9 catalase genome editing demonstrate decreased expression of catalase; (**C**) genetic inhibition of catalase expression radio-sensitized MIA PaCa-2 cells to treatment with P-AscH^−^ (5 pmol cell^−1^, 1 mM) (means ± SEM, * *p* < 0.05 vs. CRISPR control, *n* = 3); (**D**) genetic inhibition of catalase expression radio-sensitized PANC-1 cells to treatment with P-AscH^−^ (10 pmol cell^−1^, 2 mM,) (means ± SEM, * *p* < 0.05 vs. CRISPR control, *n* = 4).

Catalase is the major enzyme that converts high concentrations of H_2_O_2_ to water and oxygen [18,29,30]. Our results demonstrate that P-AscH^−^ or P-AscH^−^ plus radiation decreased the levels of catalase protein in both MIA PaCa-2 and PANC-1 PDAC cells, but not in immortalized pancreatic ductal epithelial cells H6c7 or human fetal small intestine cells, FHs74 Int (Figure 4A). Catalase activity assays were performed to corroborate these findings. In MIA PaCa-2 cells, ascorbate treatment reduced catalase activity from 2.1 ± 0.3 m*k*U to 0.8 ± 0.1 m*k*U per 10^6^ cells compared to control (means ± SEM). Radiation produced little change with a catalase activity of 2.5 ± 0.1 m*k*U per 10^6^ cells, and the combination reduced catalase activity to 1.1 ± 0.2 m*k*U per 10^6^ cells. Similar effects were seen in PANC-1 cells, with ascorbate reducing catalase activity from 9.9 ± 0.1 m*k*U to 4.9 ± 0.1 m*k*U per 10^6^ cells compared to control, radiation increasing catalase activity to 11.8 ± 0.3 m*k*U per 10^6^ cells, and the combination producing a catalase activity of 6.6 ± 0.2 m*k*U per 10^6^ cells. To further elucidate the effects of this difference in catalase in cancer cells vs. normal cells against IR or IR P-AscH^−^ treatment, we used catalase CRISPR/Cas9 KO plasmid knockouts of MIA PaCa-2 and PANC-1 cells (MIA CAT KO and PANC-1 CAT KO). Using similar protocols, control cells were generated by transfecting parental cell lines with control CRISPR/Cas 9 plasmid. Following transfection, catalase activity was reduced from 2.9 ± 0.1 m*k*U per 10^6^ cells to 1.0 ± 0.1 m*k*U per 10^6^ cells in the MIA PaCa-2 catalase knockout clone and from 6.7 ± 0.4 m*k*U per 10^6^ cells to 1.7 ± 0.1 m*k*U per 10^6^ cells in the PANC-1 clone. This is also reflected in significantly decreased protein expression, as seen in Figure 4B. Compared to RelB knockouts, MIA CAT KO and CRISPR control cells exposed to of P-AscH^−^ (5 pmol cell^−1^, 1 mM) or 1, 2, or 3 Gy with P-AscH^−^, demonstrated significant differences between the two groups (Two-way ANOVA, *p* < 0.01) (Figure 4C). The same was true when PANC-1 CAT KO and CRISPR control cells exposed to P-AscH⁻ (10 pmol cell^−1^, 2 mM) or 1, 2 3 Gy with P-AscH^−^ (Two-way ANOVA, *p* < 0.01) (Figure 4D). Taken together, these results indicate that the level of catalase plays an important role in the protection of cells against oxidative distress initiated by P-AscH^−^; catalase is the major enzyme for detoxifying high levels of H_2_O_2_ in cells [14,31].

### 3.5. Phase I Trial, Follow-Up Results

**Figure 5:**
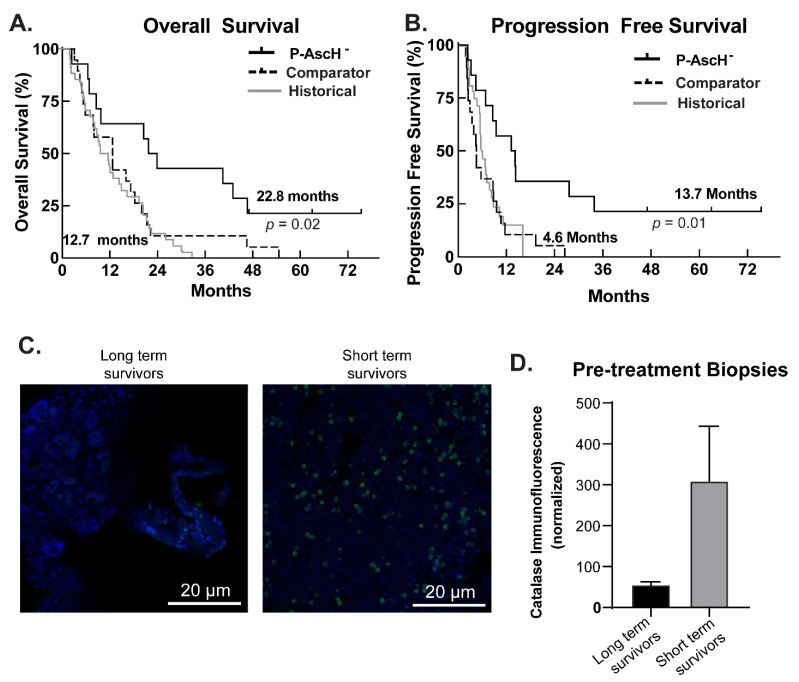
Catalase levels may predict patient outcomes after treatment with P-AscH^−^. (**A**) Overall survival from phase I trial (NCT 01049880). Kaplan–Meier curve estimating median overall survival in subjects treated with P-AscH^−^ plus gemcitabine and radiation therapy as of March 30, 2021 (*n* = 14) was 22.8 vs. 12.7 months in institutional controls treated with gemcitabine and radiation therapy (*n* = 19). (Log-Rank test *p* = 0.02). Institutional controls from the University of Iowa are equivalent to historical controls as published by Loehrer et al. [23]; (**B**) Progression free survival from phase I trial (NCT 01049880). Kaplan–Meier curve demonstrating median progression-free survival as of October 2020 in subjects treated with P-AscH^−^ plus gemcitabine and radiation therapy (*n* = 14) was 4.6 months in institutional controls treated with gemcitabine and radiation therapy vs. 13.7 months in patients receiving the same chemo-radiation therapy but also P-AscH^−^ (*n* = 19, *p* = 0.01). Institutional controls from the University of Iowa are equivalent to historical controls as published by Loehrer et al. [23]; (**C**) catalase immunofluorescence of clinical trial pre-treatment biopsy samples. Formalin-fixed, paraffin-embedded tissue samples were cut and stained with catalase antibody. DAPI was used to stain the cell nuclei; (**D**) quantification of immunofluorescence using ImageJ (*n* = 2 long-term survivors, *n* = 5 short-term survivors). Immunofluorescence was normalized to the number of cells counted per image. Pre-treatment biopsies of long-term survivors had lower levels of catalase (55.5 ± 7.5 arbitrary fluorescence units) compared to short term survivors (309 ± 134 arbitrary fluorescence units), (Means ± SEM, *p =* 0.3, Student’s *t*-test) as seen by immunofluorescence.

The trial “Gemcitabine, Ascorbate, Radiation therapy for PDAC, phase I” (NCT01852890) had 14 subjects who completed the protocol therapy between 2014 and 2017, with three long-term survivors (>6 years) with no evidence of recurrence of disease (as of 30 March 2021) resulting in a significant increase in overall survival (Figure 5A) or progression-free survival (Figure 5B). All subjects gave their informed consent for inclusion before participating in the study. The study was conducted in accordance with the Declaration of Helsinki, and the protocol was approved by the University of Iowa IRB. All three patients had received neoadjuvant FOLFIRINOX prior to starting the protocol therapy of gemcitabine, ascorbate, and radiation. All three underwent pancreaticoduodenectomy either before or after the protocol therapy. Progression-free survival in this group of long-term survivors was also significantly increased compared to historical controls [23] and comparator patients treated with the same regimen at the University of Iowa.

Of these patients, subject #2 of our trial is a female who presented in 2014 at age 65 with a 3-cm lesion in the head of the pancreas with lymphadenopathy adjacent to the pancreatic head. She was diagnosed with cT2N1M0 pancreatic adenocarcinoma with a CA-19 level of 65 U/mL at the time of diagnosis. It was determined that she had borderline resectable disease due to involvement of the first jejunal branch of the gastroduodenal artery. Standard of care chemotherapy resulted in the progression of the interface between the tumor and the adjacent superior mesenteric vein with possible involvement of the adjacent superior mesenteric vein and tight involvement of the first jejunal vein. She then enrolled in the phase I trial and completed 5 weeks of gemcitabine and radiation with P-AscH^−^. A follow-up CT scan demonstrated a slight decrease in size, and she underwent a pancreaticoduodenectomy October 2014. A biopsy revealed a well-differentiated pancreatic adenocarcinoma with marked treatment effect with microscopic rests of tumor cells, no tumor in 18 lymph nodes, with a stage of ypT3ypN0. She has had no evidence of disease recurrence as of October 2020, six years post-treatment. Her most recent CA-19-9 in April 2020 was 8 U/mL.

The second subject, subject #9, presented in January 2015 at age 62 with jaundice and fatigue. Her initial imaging demonstrated a 2.7 cm pancreatic head mass as well as regional adenopathy, portal vein invasion, and 180° abutment around the hepatic artery. She was diagnosed with stage cT3N0 pancreatic adenocarcinoma. She was determined to have a locally advanced, borderline resectable tumor due to involvement of the hepatic artery and portal vein. Due to her intolerance of neoadjuvant chemotherapy and continued concern for vascular involvement by the pancreatic mass, chemoradiotherapy was recommended. She was subsequently enrolled the phase I trial and completed 5 weeks of chemoradiotherapy with gemcitabine and P-AscH^−^. Follow-up CT one month later demonstrated no portal vein or hepatic artery involvement, and she was deemed appropriate for attempted curative resection. She underwent a pancreaticoduodenectomy demonstrating no residual viable invasive carcinoma, no tumor in 20 lymph nodes, and negative margins for a pathologic stage of ypTisN0. She has no evidence of disease recurrence on her most recent CT in May 2020.

Subject #16 was diagnosed with acute chronic pancreatitis, but also found to have a mass in the head of the pancreas. She was diagnosed with cT2N0M0 pancreatic adenocarcinoma in July of 2016 with a CA-19-9 level of 378 U/mL. Although the tumor seemed resectable at the time, neoadjuvant therapy was recommended. This was due to the increased postoperative complication risk from the presence of pancreatitis as well as high CA-19-9 level. Following chemotherapy alone, surgical resection was again postponed in order to treat newly developed portal vein and atrial thrombi. She was enrolled into the phase I trial at this point and completed the protocol therapy by January 2017. She underwent pancreaticoduodenectomy in February 2017, demonstrating multiple foci of residual adenocarcinoma with no adenocarcinoma in 31 lymph nodes, ypT2ypN0cM0. She has no evidence of disease recurrence as of June 2020, 3 years post treatment, with a CA-19-9 level of 11 U/mL.

Of the three patients, subjects #9 and #16 had pre-treatment biopsies of their tumors that were available for an assay of catalase immunofluorescence (Figure 5C). There was no remaining pre-treatment tissue for analysis for subject #2. Catalase immunofluorescence was then determined in five subjects that had shorter survival (mean 12.7 months) vs. those with long-term survival (mean 22.8 months). As seen in Figure 5D, catalase levels as measured by immunofluorescence were approximately six-fold lower in long-term survivors compared to short term survivors (Student’s *t*-test, *p =* 0.3). Though this result is not statistically significant, it may well be clinically significant as only two long-term survivors were available for analysis and demonstrated a drastic difference in comparison to short-term survivors. These data from patient biopsies also provide strong support for H_2_O_2_ to be at the center of the mechanism for the toxicity of P-AscH^−^.

## 4. Discussion

The generation of reactive oxygen species (ROS) is a major mechanism responsible for the therapeutic effect of ionizing radiation (IR) as well as nearly 50% of chemotherapeutic drugs. Currently, these therapeutic strategies are being employed to kill cancer cells without the benefit of a rational design that exploits the intrinsic differences in the cellular redox status between normal and PDAC cells. PDAC cells are under greater oxidative stress than normal cells; thus, it is thought that an additional increase in pro-oxidant levels can trigger cell death [32,33]. Therapeutic approaches that use pro-oxidants to push tumor cells into oxidative distress while stimulating adaptive responses in normal cells would be welcomed if they would enhance the effectiveness of radiation therapy.

Nrf2 is a major sensor for oxidative stress. It is a transcription factor for several antioxidant proteins such as glutamate-cysteine ligase, heme oxygenase-1, thioredoxin reductase-1, thioredoxin, and ferritin [25]. The activation of Nrf2 has been shown to protect colonic epithelial cells from IR-induced loss of cell viability [26]. Our current study demonstrates the protein activation of Nrf2 in pancreatic cancer cells and the intestinal cell lines, but not in the pancreatic ductal epithelial cell line after treatment with P-AscH^−^. with or without radiation.

The transcription factor nuclear factor-κB (NF-κB) is also subject to redox regulation. H_2_O_2_ can have both stimulatory and inhibiting roles in the functioning of NF-κB, depending on context. Wei et al. found that NF-κB transcription factor RelB is a pivotal determinant in the differential radio-sensitization effects of ascorbate in prostate cancer cells and normal epithelial cells [26]. RelB suppression decreases the expression of the sirtuin SIRT3 and MnSOD, which in turn increases oxidative and metabolic stresses in prostate cancer cells. On the contrary, ascorbate enhanced RelB expression in normal cells, improving antioxidant and metabolic defenses against radiation injury. Our current study does demonstrate a differential expression of RelB in tumor vs. normal cells (Figure 2A) after exposure to P-AscH^−^, both with and without radiation. However, clonogenic survival was not affected by the knockout of RelB in pancreatic cancer cells, suggesting a minimal role in this differential response. The different results in these two studies may be due to different tumor cell lines used and different tests of cytotoxicity (viability vs. clonogenic survival).

PDAC cells have alterations in oxidative metabolism vs. normal cells. Endogenous levels of ROS have been linked to the basic biology of PDACs. ROS are generated during normal aerobic metabolism and increased levels of these species are produced during various forms of oxidative stress. The net intracellular concentration of ROS is the result of the production of ROS and the ability of antioxidant systems to remove them. The induction or inhibition of proliferation due to oxidative stress is dependent on levels of oxidants in the cell. Low levels of oxidants can stimulate proliferation while higher levels inhibit [34]. Increases in levels of nitrotyrosine, a footprint for peroxynitrite, a potent reactive nitrogen/oxygen species formed by the reaction of superoxide with nitric oxide, has been demonstrated in pancreatic cancer specimens compared to normal pancreases [35]. Normal cells with a more robust antioxidant system than PDAC cells will be resistant to P-AscH^−^ induced oxidative distress.

Previous studies from our group have demonstrated that the differential sensitivity to P-AscH^−^ across PDACs was strongly correlated with their individual capacities to remove H_2_O_2_ [7,8]. Other groups have also demonstrated the importance of catalase in the role of P-AscH^−^-induced toxicity [32,36] and methods of increasing P-AscH^−^ toxicity in chemotherapeutic resistant cells by silencing catalase [37]. In general, the rate constants for the removal of extracellular H_2_O_2_ generated from P-AscH^−^ are approximately two-fold higher in normal cells than in cancer cells studied in vitro [18]. Our current study suggests that catalase levels may also play a role in the response to P-AscH^−^-induced chemo-radio-sensitivity in PDAC in vivo. In pre-treatment tissue biopsies from patients with locally advanced PDAC who underwent gemcitabine and radiation therapy, catalase levels were anticorrelated with progression-free survival and overall survival. These findings correlate well with our previous studies, demonstrating that the response to P-AscH^−^ in murine models of PDAC paralleled the in vitro results when PDAC cells with varying catalase levels were exposed to P-AscH^−^ [18].

## 5. Conclusions

P-AscH^−^ in conjunction with radiation resulted in similar changes in Nrf2, RelB, and cellular bioenergetics in PDAC cells and normal cells. Changes in catalase levels resulted in P-AscH^−^ radio-sensitization in PDAC. Our data from a phase I clinical trial suggest that catalase levels in the primary tumor may play a role in the response to P-AscH^−^ radio-sensitization. Thus, catalase levels could be a possible indicator on how tumors might respond to P-AscH^−^.

## Data Availability

Not applicable.
